# Dietary intake of pistachios or mixed nuts results in higher systemic antioxidant capacity with minimal effects on bone in adolescent male rats

**DOI:** 10.1017/jns.2022.121

**Published:** 2023-01-24

**Authors:** Brooke E. Wickman, Zachary S. Clayton, Eric Rochester, Mark Kern, Mee Young Hong, Changqi Liu, Shirin Hooshmand

**Affiliations:** 1 School of Exercise and Nutritional Sciences, San Diego State University, 5500 Campanile Drive, San Diego, CA 92182, USA; 2 Department of Integrative Physiology, University of Colorado Boulder, 354 UCB, Boulder, CO 80309, USA

**Keywords:** Antioxidants, Bone, Mixed nuts, Osteogenesis, Oxidative stress

## Abstract

Nutrition is a key determinant of bone health and attainment of peak bone mass. Excess oxidative stress induces bone loss while increasing antioxidant capacity promotes protective effects on bone. Nuts are rich in antioxidants; therefore, we tested the hypothesis that compared to a control diet high in fat (40 % energy) and cholesterol, diets containing isocaloric amounts of pistachios (8·1 % g/g) or mixed nuts (7·5 % g/g) for 8 weeks would result in greater bone health in male adolescent (3 weeks; a state of continued skeletal growth) Sprague-Dawley rats. We found no difference in bone mechanical properties among groups. Tibial apparent density was ~5 % higher in the pistachio and mixed nuts groups *v*. control (*P* < 0·05) with no clear difference detected for the femur. Expressions of genes known to impact bone turnover and serum bone turnover biomarkers were unaffected by either diet relative to control. Serum antioxidant capacity was ~2-fold higher in the pistachio and mixed nuts groups compared with control (*P* < 0·05) but were similar between groups. Therefore, pistachios and mixed nuts may increase tibial density, in part, due to increasing antioxidant capacity. Longer dietary interventions may be necessary to elicit detectable changes in other bones (e.g. femur) and to detect potential mechanisms for the possible bone protective effects of nuts.

## Introduction

In the United States, approximately two million males have osteoporosis^(1)^; however, much of the literature is focused on osteoporosis in females. Furthermore, osteoporotic fractures are a leading cause of morbidity and mortality in aging males^(2)^. As such, accretion of the highest peak bone health in adolescence and young adulthood may protect against and/or delay age-associated bone loss^(3)^. Nutrition plays a key role in bone metabolism and, during growth, achievement of peak bone mass^(4)^. Thus, implementing nutrition interventions early in life may be a therapeutic strategy to achieve greater bone health in later years.

Nuts have a diverse nutrient profile and are high in vitamins, minerals and phenolic compounds^(5)^ that may be effective at promoting bone health via activation of osteoblasts or suppression of osteoclast activity, which may in part be attributed to antioxidant effects^(5)^. Nuts also contain fibre, protein, calcium, magnesium, potassium, folic acid, niacin, vitamin B_6_ and vitamin K^(5,6)^, which are all associated with the maintenance and development of bone^(4,7)^. However, to take advantage of a broader nutrient profile, consumption of mixed nuts may prove to be more effective at enhancing indices of bone health compared with a single nut. To date, there have not been any studies assessing the effect of mixed nuts on indices of bone health or if mixed nuts are more effective than a single nut.

A potential mechanism underlying bone resorption is excess oxidative stress without an adequate compensatory increase in antioxidant defenses^(8)^. Enhancing antioxidant capacity has been shown to promote bone health in the setting of advanced age (a timeframe associated with significant bone loss)^(8)^, but the relation of antioxidant status and bone health in adolescents is not currently known. Notably, nuts are rich in antioxidants^(9)^. As such, dietary supplementation with nuts may enhance bone health early in life, in part due to increasing antioxidant capacity.

The purpose of the present study was to investigate the effect of a single nut (pistachios, a nut shown to increase antioxidant status and reduce oxidative stress in humans^(10)^) and a blend of commonly consumed nuts on bone health, including bone density, mechanical properties, osteogenic gene expression, circulating markers of bone growth and turnover, bone resorption and antioxidant capacity in young male rats relative. We hypothesised that rats consuming a pistachio-enriched diet and diet enriched with mixed nuts (both diets mixed within a high fat [40 % energy]/high cholesterol diet that was selected to allow for assessment of separate outcomes on lipid metabolism) would have better bone health relative to rats consuming the high fat/high cholesterol diet alone, and that the better bone health would be augmented in the group consuming mixed nuts *v*. pistachios.

## Materials and methods

### Animal and diets

All procedures were performed in strict accordance with the protocol approved by the Animal Care and Use Committee at San Diego State University (APF 18-01-001H) and in accordance with the guidelines outlined for the care and use of laboratory animals^(11)^. Male Sprague-Dawley rats (*n* 30) were purchased at 3 weeks of age from Harlan Industries (Indianapolis, IN, USA) for use in this study. Rats were given 3–4 d to acclimate to their environment before dividing rats into three groups of ten and assigning to isocaloric diets consisting of control high fat/high cholesterol diet, control diet with 8·1 % pistachio w/w, or control diet with 7·5 % mixed nut w/w diet for 8 weeks, as described previously^(12)^. Nuts account for ~9·8 % of total kcals for the pistachio diet or mixed nut diet. Nut dosages were chosen to provide a similar percentage of total kcals to human studies^(13)^. All ingredients except nuts were purchased from Dyets (Bethlehem, PA, USA). Pistachios were obtained from The Wonderful Company (Lost Hills, CA, USA). Mixed nuts, peanuts and walnuts were Kirkland brand (Issaquah, WA, USA). The mixed nuts contained almonds, Brazil nuts, cashews, macadamia nuts and pecans. Because some commonly consumed nuts were absent from this product, 1·125 g walnuts (15 %), 1·125 g pistachios (15 %) and 1·5 g peanuts (20 %) were added per 3·75 g of the basic mixture (50 %). All diets consisted of 42 % carbohydrate, 18 % protein and 40 % fat as percentages of energy provided. The composition of each diet is provided in Table 1. Rats were singly housed in cages within an environmentally controlled animal care laboratory with a 12:12 h light/dark cycle and provided with *ad libitum* access to both feed and standard drinking water. Body weight was measured weekly throughout the study, while food and water intake were measured at weeks 3, 5 and 7. Animal health checks were conducted daily by the veterinary or research staff.

**Table 1.** Diet composition of LabDiet #5001 (g/1000 g)

Ingredient	Control	Pistachios	Mixed nuts
Cornstarch	143·00	134·00	135·00
Sucrose	330·00	324·00	327·00
Cellulose	50·00	37·00	42·00
Casein	200·00	183·00	187·00
Corn oil	50·00	50·00	50·00
Milk fat	160·00	124·00	117·00
Cholesterol	10·00	10·00	10·00
DL-Methionine	3·00	3·00	3·00
Sodium cholate	5·00	5·00	5·00
Choline bitartrate	4·00	4·00	4·00
Pistachios	0·00	81·00	0·00
Mixed nuts	0·00	0·00	75·00
Vitamin mix^a^	10·00	10·00	10·00
Mineral mix^b^	35·00	35·00	35·00
**Total**	**1000·00**	**1000·00**	**1000·00**

a Composition of vitamin mix (g/kg mixture): Carotene 23·0; menadione sodium bisulphite (source of vitamin K activity) 125·0; Thiamine hydrochloride 160·0; Riboflavine 65·0, Niacin 120·0; Pantothenic acid 44·0; Choline chloride 225·0; Folic acid 17·0; Pyridoxine 60·0; D-biotin 30·0; vitamin B12 50·0; Vitamin A acetate 10·0; D activated animal sterol (source of vitamin D3 activity) 38·0; DL-alpha tocopheryl acetate (source of vitamin E activity) 33·0.

b Composition of mineral mix (g/kg mixture): Fluorine 150·0; Iron 57·0; Zinc 75·0; Manganese 70·0; Copper 13·0; Cobalt 9·0; Iodine 1·0; Chromium 12·0; Selenium 20·0; Chlorine 67·0; Sodium 90·0; Sulphur 46·0, Magnesium 41·0, Potassium 118·0, Phosphorus (non-phytate) 59·0; Phosphorus 76·0, Calcium 96·0.

At the conclusion of the study, animals were anesthetised by CO_2_ exposure and subsequently euthanized via decapitation for exsanguination. Blood was allowed to clot and centrifuged for 10 min at 1200×*g* at 4°C. Serum was stored at −80°C until time of analysis. Right femurs and tibiae were dissected, cleaned of muscle and connective tissue, and snap frozen in liquid nitrogen and stored at −80°C. Left femurs and tibiae were dissected, wrapped in saline (0·9 % NaCl) soaked gauze and stored at −20°C.

### Analysis of apparent bone density

Left femurs and tibiae were first hydrated in distilled water at reduced atmospheric pressure (360 mmHg) for 1 h. After hydration, the weights of bones were measured both submerged in and out of water using the Mettler Toledo Density Kit ME-DNY-4 (Columbus, OH, USA). Bone densities (g/cm3) were determined using Archimedes’ principle and calculated by the following formula, as we have previously described^(14)^:
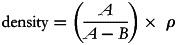
where *A* is the weight of hydrated bone weighed out of water, *B* is the weight of hydrated bone submerged in water and *ρ* is the density of distilled water at 24°C.

### Analysis of bone mechanical properties

Left femurs were thawed at room temperature, fully hydrated in distilled water for 1 h under reduced atmospheric pressure (360 mmHg), and measured by calliper to ascertain dimensions for length, width and height. A TA.XT Plus Texture Analyzer (Texture Technologies Corp. and Stable Micro Systems, Ltd., Hamilton, MA, USA) with a 50 kg load cell and a three-point bending rig was used to analyse texture and mechanical properties of bone as an estimation for diaphysis strength and fracturability. Supported horizontally at both ends with a gap distance of 20 mm, femurs were placed with the posterior side facing down. A 3 mm width blade travelling 15 mm downwards at 1 mm/s with 0·049 N trigger force compressed and fractured the bones on the anterior side at the exact midpoint between the supports located at the proximal and distal ends of the bone. Force and displacement data, including ultimate load (N), displacement-to-ultimate (mm), stiffness (N/mm) and energy-to-ultimate (mJ) were recorded at 200 Hz by the Exponent software (Version 6, 1, 10, 0; Stable Micro System; Godalming, UK). In order to account for size variation in samples, Young's modulus of bend (GPa) was calculated as follows:
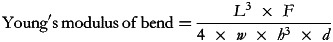
where *L* is the bone length, *F* is the breaking force, *w* is the bone width, *h* is the bone thickness and *d* is the breaking distance.

### Biochemical analyses

Serum total antioxidant capacity (Cayman, Ann Arbor, MI, USA), osteocalcin (Immunotopics, Inc., San Clemente, CA, USA) and deoxypyridinoline (Quidel, San Diego, CA, USA) were assessed according to manufacturers’ guidelines.

### Gene expression

Right femoral heads were used for RNA extraction. TRI Reagent® solution (Sigma-Aldrich, Bellefonte, PA, USA) was added to the femoral heads before grinding samples into a fine powder using SPEX 6700 freezer mill (SPEX Sample Prep, Metuchen, NJ, USA). RNA purity and concentration were measured using absorbance at A260 nm and A260/280 nm ratio using a Nano-Drop 1000A Spectrophotometer (Fisher Scientific LLC, Hanover Park, IL, USA).

Reverse transcription of femoral RNA to cDNA was performed using an iScript cDNA Synthesis Kit according to the manufacturer's protocol. Genes of interest included TNF superfamily member 11 (*Tnfsf11*), TNF receptor superfamily member 11A (*Tnfrsf11a*), TNF receptor superfamily member 11B (*Tnfrsf11b*), NADPH oxidase 4 (*Nox4*), bone gamma-carboxyglutamate protein (*Bglap*), sclerostin (*Sost*), dickkopf WNT signalling pathway inhibitor 1 (*Dkk1*), 5′ nucleotidase, ecto (*Nt5e*), Wnt family member 3A (*Wnt3a*), axin 1 (*Axin1*), runt-related transcription factor 2 (*Runx2*) and LDL receptor-related protein 5 (*Lrp5*). Genes were normalised to the housekeeping gene glyceraldehyde-3-phosphate-dehydrogenase (*Gapdh*). For each gene being investigated, a master-mix consisting of nuclease free water, the corresponding forward and reverse primer, and SsoAdvanced Universal SYBR Green Supermix was prepared. cDNA samples were then diluted to a concentration of 230 ng/μl^(1)^ based on calculations enabling two technical replicates. In a 384-well plate, 1 μl of diluted cDNA and 9 μl of corresponding master-mix were added per well. After covering with MicroAMP Optical Adhesive Film and centrifuging at 1000 rpm for 1 min, the samples were loaded into a ViiA Real-Time quantitative polymerase chain reaction (qPCR) Detection System set (Applied Biosystems, Foster City, CA, USA) for 40 cycles. Each cycle was performed under the following conditions: denaturation of cDNA strands at 95°C for 15 s and annealing of the primer and extension of the complementary DNA at 60°C for 30 s. Calculation of the Ct values and analysis of gene expression amplification plots was performed with the ViiA™ 7 Software Version 1.1. Differences in gene expression between diet groups were calculated using the delta-delta-CT method.

### Statistical analysis

Data were assessed for outliers using the Grubb's test (Graph Pad online calculator) prior to statistical analysis with an exclusion threshold of 0·05. After confirming normality, one-way ANOVAs were used to analyse data and Bonferroni multiple comparisons *post hoc* tests were used as necessary. Significance was set at *α* = 0·05. All data are presented as means ± sem. Statistics were calculated using Graph Pad Prism version 9 (Graph Pad Software, La Jolla, CA, USA).

## Results

### Body weight, energy intake and water consumption

There were no differences in food or water intake among groups^(12)^. Initial body weight and body weight at the time of sacrifice were not different among groups (Table 2). As reported previously^(12)^, no differences in weights of liver, spleen or epididymal fat pads were detected.

**Table 2.** Initial and final body weight in young adult male rats consuming a control diet or diet containing pistachios or mixed nuts for 8 weeks

	Control	Pistachios	Mixed nuts	*P-*value
Initial body weight (g)	49·3 ± 1·1	49·8 ± 0·9	49·9 ± 0·8	0·89
Final body weight (g)	369·4 ± 4·5	377·6 ± 5·0	378·3 ± 7·9	0·51

Data are expressed as mean ± sem. *P-*value represents the result of a one-way ANOVA.

### Apparent bone density

**Fig. 1.**
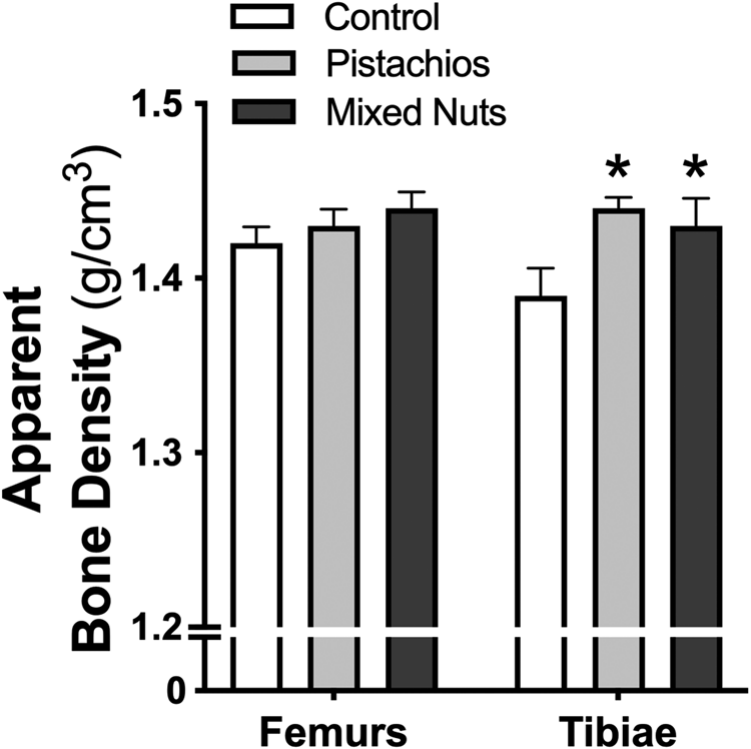
Control diet *v*. a pistachio-enriched diet *v*. a mixed nuts-enriched diet on apparent bone density of the femurs and tibiae. *P < 0·05 *v*. control within bone type.

There was a significant difference among groups for tibial apparent density (*P* = 0·02). *Post hoc* analyses indicated that tibial apparent density of the pistachio group (*P* = 0·01) and the mixed nuts group (*P* = 0·04) was greater than control; however, there was no difference between the pistachio and mixed nuts groups (*P* = 0·56). Femoral apparent density was not different among groups (Fig. 1).

### Mechanical properties of bone

There were no differences in ultimate force, displacement-to-ultimate, energy-to-ultimate, Young's modulus of bend or stiffness between groups for the femur or the tibia (Tables 3 and 4).

**Table 3.** Femoral weight, length, thickness, width and mechanical bone properties in young adult male rats consuming a control diet or diet containing pistachios or mixed nuts for 8 weeks

	Control	Pistachios	Mixed nuts	*P-*value
Dry weight (g)	0·92 ± 0·02	0·90 ± 0·02	0·90 ± 0·01	0·43
Length (cm)	3·60 ± 0·02	3·61 ± 0·03	3·60 ± 0·03	0·91
Thickness (mm)	0·34 ± 0·01	0·32 ± 0·02	0·32 ± 0·01	0·96
Width (mm)	0·53 ± 0·02	0·53 ± 0·01	0·60 ± 0·02	0·26
Ultimate load (N)	116·99 ± 3·58	122·10 ± 5·61	113·79 ± 3·43	0·40
Displacement-to-ultimate (mm)	1·63 ± 0·11	1·51 ± 0·08	1·65 ± 0·12	0·72
Energy-to-ultimate (mJ)	74·31 ± 4·29	81·72 ± 3·60	72·12 ± 5·19	0·29
Young's modulus (GPa)	62 671·13 ± 7906·37	69 663·01 ± 4467·53	57 081·03 ± 4379·58	0·32
Stiffness (N/mm)	74·31 ± 4·29	81·72 ± 3·60	72·12 ± 5·19	0·28

Data are expressed as mean ± sem.

**Table 4.** Tibial weight, length, thickness, width and mechanical bone properties in young adult male rats consuming a control diet or diet containing pistachios or mixed nuts for 8 weeks

	Control	Pistachios	Mixed nuts	*P-*value
Dry weight (g)	0·79 ± 0·03	0·77 ± 0·02	0·74 ± 0·02	0·77
Length (cm)	3·89 ± 0·05	3·86 ± 0·02	3·83 ± 0·03	0·86
Thickness (mm)	0·37 ± 0·02	0·31 ± 0·01	0·34 ± 0·02	0·74
Width (mm)	0·47 ± 0·02	0·41 ± 0·01	0·44 ± 0·02	0·88
Ultimate load (N)	70·45 ± 3·32	72·53 ± 3·39	76·62 ± 2·88	0·64
Displacement-to-ultimate (mm)	1·88 ± 0·13	2·49 ± 0·53	2·18 ± 0·38	0·61
Energy-to-ultimate (mJ)	38·45 ± 1·87	34·88 ± 3·49	40·72 ± 4·26	0·22
Young's modulus (GPa)	26 592·88 ± 4151·51	45 399·56 ± 5470·49	38 331·99 ± 6356·94	0·50
Stiffness (N/mm)	38·45 ± 1·87	34·88 ± 3·49	40·72 ± 4·26	0·22

Data are expressed as mean ± sem.

### Serum biomarkers of bone turnover and antioxidant capacity

**Fig. 2.**
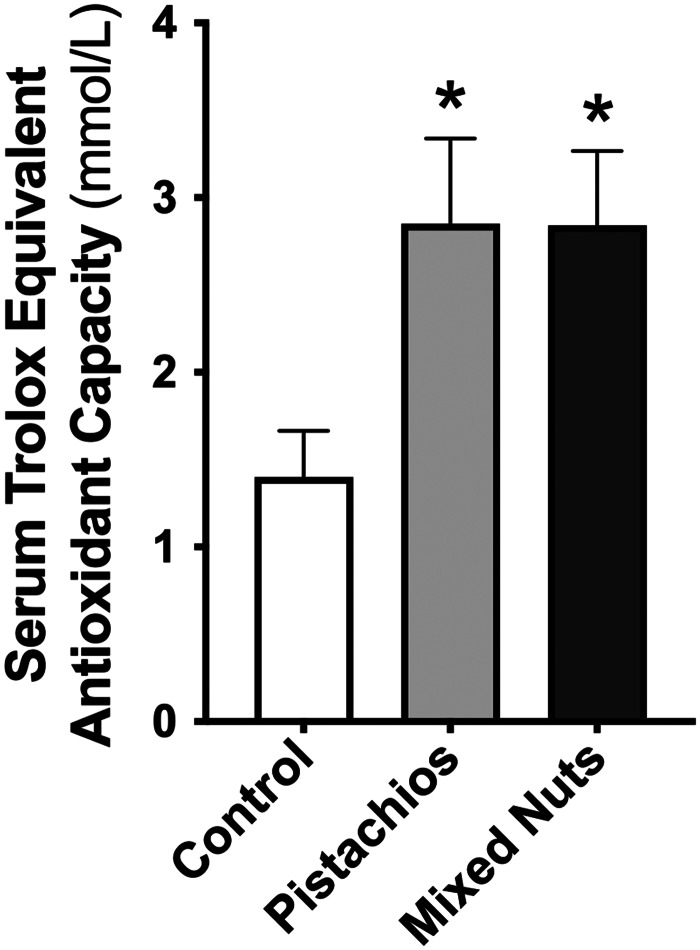
Control diet *v*. a pistachio-enriched diet *v*. a mixed nuts-enriched diet on serum total antioxidant capacity. *P < 0·05 *v*. control.

There were no differences in serum osteocalcin or deoxypyridinoline between groups (Table 5). There was a significant difference among groups for serum total antioxidant capacity (*P* = 0·03). Relative to the control group, serum total antioxidant capacity was higher in the pistachio group (*P* = 0·04) and the mixed nuts group (*P* = 0·04) (Fig. 2); however, there was no difference between the pistachio group and mixed nuts group (*P* = 0·99).

**Table 5.** Circulating concentrations of osteocalcin and deoxypyridinoline in young adult male rats consuming a control diet or diet containing pistachios or mixed nuts for 8 weeks

	Control	Pistachios	Mixed nuts	*P-*value
Osteocalcin (ng/ml)	20·6 ± 1·8	22·4 ± 2·4	19·1 ± 2·2	0·55
Deoxypyridinoline (ng/ml)	14·2 ± 0·4	16·7 ± 1·4	16·1 ± 0·5	0·15

Data are expressed as mean ± sem.

### Osteogenic gene expression

There were no differences in osteogenic gene expression among groups (Table 6).

**Table 6.** Fold change and relative significance of osteogenic gene expression between young adult male rats consuming a control diet or diet containing pistachios or mixed nuts for 8 weeks

Gene	Pistachios (fold change *v*. control)	*P*-value	Mixed nuts (fold change *v*. control)	*P*-value
*Tnfsf11*	1·71	0·07	1·07	0·19
*Tnfsf11a*	1·03	0·73	1·02	0·81
*Tnfsf11b*	1·01	0·87	1·10	0·25
*Nox4*	0·98	0·90	0·95	0·55
*Bglap*	1·06	0·27	1·01	0·81
*Sost*	0·99	0·66	1·04	0·48
*Dkk1*	0·99	0·53	1·03	0·39
*Nt5e*	1·00	0·93	1·02	0·46
*Wnt3a*	1·05	0·20	1·07	0·10
*Axin1*	0·99	0·89	1·05	0·48
*Runx2*	1·04	0·51	1·05	0·34
*Lrp5*	0·93	0·33	0·94	0·28

TNF superfamily member 11 (*Tnfsf11*), TNF receptor superfamily member 11A (*Tnfrsf11a*), TNF receptor superfamily member 11B (*Tnfrsf11b*), NADPH oxidase 4 (*Nox4*), bone gamma-carboxyglutamate protein (*Bglap*), sclerostin (*Sost*), dickkopf WNT signalling pathway inhibitor 1 (*Dkk1*), 5′ nucleotidase, ecto (*Nt5e*), Wnt family member 3A (*Wnt3a*), axin 1 (*Axin1*), runt-related transcription factor 2 (*Runx2*) and LDL receptor-related protein 5 (*Lrp5*). *n* 10/group.

## Discussion

Our major findings are that consumption of pistachios and mixed nuts resulted in higher tibial bone density and serum antioxidant capacity compared with a nut-free high fat/high cholesterol diet. No other impacts on measures of bone density or bone mechanical properties were detected, nor were any effects on bone biomarkers or the expression of genes known to regulate bone metabolism. Furthermore, in contrast to our hypothesis, mixed nuts failed to produce any augmented changes in comparison to a single nut (pistachios). A longer dietary intervention may have elicited other changes in bone health parameters that we failed to detect in this 8-week study. Overall, these findings provide scant new evidence regarding the influence of dietary nut consumption on indices of bone health but suggest a need to obtain potentially translational evidence regarding nut consumption and bone health in humans.

Although recent data suggest that markers of bone turnover may help to better predict fracture risk in the elderly when used in combination with other markers such as bone density^(15)^, the observed changes in tibial whole bone density with pistachio and mixed nut consumption are of potential importance if confirmed by future research, since an increase in bone density early in life may delay the onset of age-related bone loss^(16)^. Specifically, age-related bone loss is predicted to be delayed by 13 years for every 10 % increase in bone density early in life^(16)^. Although tibial fractures are less common in the elderly relative to femoral fractures, tibial fractures do often occur and are associated with age-related frailty^(17)^.

Excess oxidative stress accompanied by an inadequate compensatory increase in antioxidant enzyme defenses promotes bone loss^(8)^. Thus, increasing antioxidant status in early life (and maintaining levels into late life) may be a therapeutic strategy for delaying age-related bone loss. Mechanistically, antioxidants can attenuate the production of osteoclasts and promote differentiation of osteoblasts^(8)^. However, we did not detect any changes to support such effects. Other research has demonstrated that the antioxidant lycopene can suppress osteoclastogenesis^(18)^, but supporting data were measured *in vitro* in cultured osteoclasts.

The amount of pistachios and mixed nuts used in the present study was chosen to provide a percentage of total kcals that is similar to that utilised in human studies that have fed nuts^(13)^. Although we detected a modicum of favourable improvements in indices of bone health with these doses that was consistent for both pistachios and mixed nuts, our findings do not clearly support the use of dietary nut consumption as a therapeutic approach since the study was conducted in a rodent model and any potential benefits were limited to the apparent bone density of the tibia. However, some other evidence does exist supporting a potential benefit of nut consumption for bone health. A retrospective analysis which assessed the influence of habitual nut consumption on bone mineral density in post-menopausal women revealed that nut consumption was associated with greater bone mineral density^(19)^. Since the results of that study and our study should be viewed as preliminary, long-term clinical trials are needed to confirm the translational potential of nut consumption for improving bone health in both females and males.

There are several possible limitations in the present study. First, the duration of the study was relatively short for bone-targeted intervention studies, so we may have observed changes in femoral bone density and/or other indicators of bone health with a longer intervention. Second, we were not able to specifically assess hip and vertebral bone density, which is a common fracture site in older adults^(20)^. Third, we were unable to assess tibial osteogenic gene expression (too low of total RNA yield), which may have provided insight into the observed site-specific differences in bone density. Fourth, the background diet was a high fat/high cholesterol diet; thus, future studies should consider using a more typical lower fat rodent diet to ensure that these effects are generalisable to other diets. Lastly, we did not assess bone microarchitecture, which would have informed whether pistachios and mixed nuts influence bone quality.

Overall, we have shown that physiologically relevant doses of pistachios and mixed nuts increased tibial whole bone density and systemic antioxidant capacity but failed to demonstrate other impacts on bone health or to determine if tibial differences occurred through enhanced bone formation or reduced resorption mechanisms. The potential effects on the tibia could provide the basis for forward translation to humans in order to determine the influence of dietary nut consumption on bone density and markers of bone health. If shown to be effective in humans, consumption of nuts could be recommended as a therapeutic strategy for increasing bone density in adolescent and young adult years, and therefore attenuating the rate of age-related bone loss.
